# Beyond Hyperexcitability: A Review of Neural Mechanisms in Charles Bonnet Syndrome

**DOI:** 10.3390/neurosci7020031

**Published:** 2026-03-03

**Authors:** Eric Altieri, Luca Battaglini

**Affiliations:** 1 Neuro.Vis.U.S. Laboratory, University of Padova, 35131 Padova, Italy; eric.altieri@unipd.it; 2 University Centre for Clinical Psychological Services (SCUP), University of Padova, 35131 Padova, Italy; 3 Department of General Psychology, University of Padova, 35131 Padova, Italy

**Keywords:** Charles-Bonnet syndrome, release phenomena, cortical hyperexcitability, predictive processing theory

## Abstract

Charles Bonnet syndrome (CBS) is characterized by complex visual hallucinations in visually impaired individuals who maintain intact cognitive function. Despite significant progress in understanding this condition, the precise neural mechanisms underlying CBS remain incompletely understood. This review synthesizes current evidence regarding the pathophysiology of CBS, with particular emphasis on emerging neurobiological models that extend beyond simple cortical hyperexcitability. Recent neuroimaging, neurophysiological, and computational modeling studies suggest that CBS hallucinations may arise from complex interactions among deafferentation-induced neural plasticity, neurotransmitter imbalances, and altered functional connectivity within visual processing hierarchies. The evidence increasingly points toward a model involving desynchronization between bottom-up and top-down visual processing pathways, rather than mere hyperexcitability of deafferented visual cortex. This integrated perspective has important implications for both the theoretical understanding of visual perception and the development of targeted therapeutic interventions.

## 1. Introduction

The history of Charles Bonnet syndrome (CBS) begins in 1760 with the publication of “Essai analytique sur les facultés de l’âme,” a book written by the Swiss naturalist, philosopher, and biologist Charles Bonnet. In what some consider the first scientific publication on hallucinatory experiences [1], Bonnet described the visual phenomena experienced by his grandfather, Charles Lullin. Lullin, an 89-year-old magistrate, began perceiving unusual visual perceptions following cataract surgery: men, women, birds, and buildings of variable dimensions and forms, none of which were evoked by external stimuli. Importantly, Lullin maintained full consciousness and was aware that these visions were not real but rather creations of his mind [2,3]. Interestingly, it was not Charles Bonnet who named this syndrome after himself, but rather his compatriot Georges de Morsier [4], who later used Bonnet’s name in recognition of his being the first to describe the condition. The phenomenon of visual hallucinations following visual loss has been documented throughout medical history. In a compelling case reported by Cohn in his scientific article “Phantom Vision” [5], a man who lost his left eye in an explosion and underwent surgical removal of the entire ocular bulb subsequently experienced visual sensations of malformed clouds emanating from the now-empty orbit. Cohn documented seven individuals who, after partial or complete vision loss, began experiencing visual sensations from their missing or non-functioning eyes. Although Cohn did not use the term, these individuals were likely experiencing Charles Bonnet syndrome [5].

CBS is defined by the presence of complex visual hallucinations in individuals with visual impairment who retain intact cognitive function and insight into the unreality of their hallucinatory experiences [1,6]. These hallucinations can range from simple geometric patterns to elaborate scenes involving people, animals, and landscapes. The syndrome has garnered renewed scientific interest in recent years for several reasons: first, the aging population and increased prevalence of age-related visual impairment have led to greater clinical recognition of CBS [7]; second, CBS offers a unique window into visual processing mechanisms in the brain [8]; and third, the condition has been recently included in the 11th revision of the International Classification of Diseases (ICD-11), facilitating more standardized diagnosis and research [9]. Despite this increased attention, CBS remains underdiagnosed and often poorly understood by healthcare professionals [10]. Furthermore, the exact neurobiological mechanisms underlying visual hallucinations in CBS continue to be debated.

This review was motivated by the clinical imperative to develop a more comprehensive understanding of CBS patients beyond isolated theoretical frameworks. Despite significant advances in specific domains of CBS research, clinicians face challenges in translating compartmentalized mechanistic models into holistic patient assessment and management. The fragmentation of explanatory models across ophthalmological, neurological, and psychiatric perspectives has created barriers to integrated care. Visual hallucinations in CBS represent a complex phenomenon that transcends traditional disciplinary boundaries, necessitating a synthesis of diverse evidence to inform clinical practice.

To address this clinical need, we synthesized evidence from multiple disciplines to develop an integrated understanding of CBS pathophysiology. We conducted a comprehensive literature search using PubMed, Web of Science, and Google Scholar databases for articles published between 1980 and 2025, with particular emphasis on research from the past two decades. Search terms included “Charles Bonnet syndrome,” “visual hallucinations,” “deafferentation,” “cortical hyperexcitability,” “visual impairment hallucinations,” “predictive processing hallucinations,” and “neural desynchronization.” We prioritized peer-reviewed empirical studies, computational models, neuroimaging investigations, and clinical reports that contributed to understanding CBS mechanisms. Additionally, we identified relevant articles through citation tracking from seminal papers.

By developing a unified conceptual framework that bridges multiple levels of explanation, from cellular mechanisms to network dynamics to phenomenological experience, this review aims to facilitate more nuanced clinical reasoning and potentially guide more targeted therapeutic approaches. While not claiming exhaustive coverage of all CBS literature, this review encompasses major theoretical frameworks and empirical findings that inform current understanding of CBS pathophysiology, addressing an unmet need in clinical settings where practitioners must consider the multifaceted nature of visual hallucinations when evaluating and treating patients with CBS.

To achieve this goal, we have organized the review around an integrated multilevel framework. We begin by examining the epidemiology and clinical features of CBS, establishing its prevalence, risk factors, and distinctive hallucination characteristics. We then explore mechanistic explanations, starting with deafferentation as the initial trigger, followed by computational evidence that illustrates how the brain responds to sensory deprivation. Next, we move beyond simple hyperexcitability models to discuss how neural desynchronization across visual processing hierarchies disrupts normal predictive processing. We integrate these perspectives into a cohesive model that explains the complex phenomenology of CBS hallucinations. We then examine the clinical implications of this integrated model for diagnosis and treatment approaches, discussing both pharmacological and non-pharmacological interventions. Finally, we outline promising avenues for future research that could further advance our understanding of this fascinating condition and lead to improved therapeutic strategies.

## 2. Epidemiology and Clinical Features

### 2.1. Prevalence and Risk Factors

The reported prevalence of CBS varies considerably across studies and patient populations. A comprehensive meta-analysis found an overall pooled prevalence of 10.2% (95% CI: 7.2–14.1%) among ophthalmic patients, with the highest rates observed in vision rehabilitation patients (24.6%) [11]. In populations with significant visual impairment, Subhi et al. [12] reported a pooled prevalence of 19.7% (95% CI: 13.8% to 26.4%). Based on global estimates of visual impairment, they calculated that approximately 47.2 million people worldwide may experience CBS. The prevalence of CBS appears to vary by underlying condition. In patients with glaucoma, prevalence rates ranged from 2.8% (95% CI: 0.7% to 6.1%) in university clinic patients to 20.1% (95% CI: 16.8% to 23.6%) in those attending vision rehabilitation centers [12]. For patients with retinal diseases, including age-related macular degeneration (AMD), prevalence rates typically range between 11.8% and 17.7% [11].

Several risk factors for CBS have been identified across multiple studies. Visual acuity below 0.3 in the best eye appears to be significantly associated with CBS development [13], with bilateral visual impairment posing a greater risk than unilateral impairment. Advanced age consistently emerges as an independent risk factor, with significantly higher prevalence rates observed in patients over 64 years [11,13]. Female sex has been identified as a potential risk factor in several studies. Christoph et al. [11] found a statistically significant association between female gender and CBS (95% CI: 1.29–3.34), though earlier studies such as Teunisse et al. [11,13] did not confirm this association. Other proposed risk factors include reduced contrast sensitivity and social isolation [12]. Notably, the specific underlying eye disease appears less important than the degree of visual impairment itself. Teunisse et al. [13] found little difference in CBS prevalence across common ophthalmic diagnoses, concluding that low visual acuity was more strongly associated with the syndrome than specific eye diseases.

### 2.2. Hallucination Clinical Features

**Figure 1:**
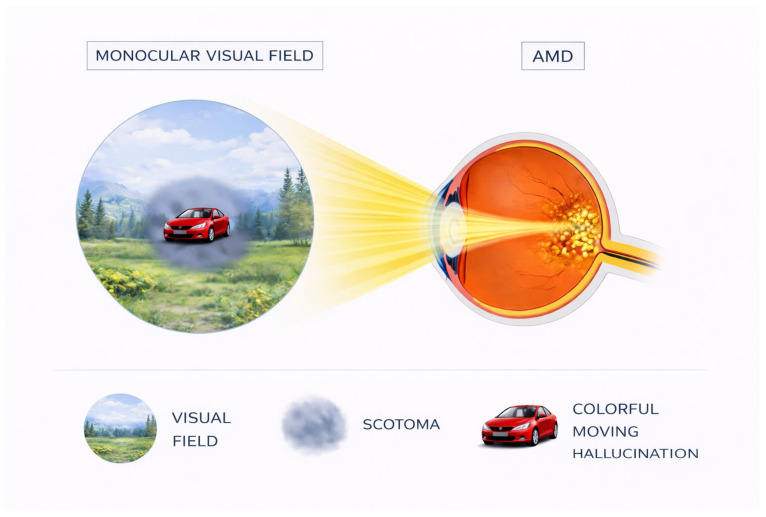
Representation of the monocular visual field in age-related macular degeneration, highlighting the occurrence of visual hallucinations within the central scotoma.

Charles Bonnet syndrome is characterized by complex, formed visual hallucinations occurring in the context of visual impairment. The American Psychiatric Association defines hallucinations as “perception-like experiences that have the clarity and impact of a real perception but without the external stimulation of the relevant sensory organ. Hallucinations must be distinguished from illusions, in which an external stimulus is misperceived or misinterpreted”. These hallucinations are classified as “complex” because they contain structured images such as people, animals, objects, or geometric patterns, distinguishing them from elementary hallucinations consisting of lights, flashes, or sparkles [8,14,15]. The hallucinations are predominantly visual and appear in the area of visual field loss, though rare cases with concurrent auditory components have been documented [16]. Chromatic properties are notable, with 72% of hallucinations presenting in color [15,17]. Movement is present in approximately 63% of hallucinations, and 85% appear directly in the central visual field [15], as shown in Figure 1. An important characteristic is that hallucinatory images appear clear and well-defined to patients despite their visual deficits [18], suggesting intact higher visual processing despite impaired sensory input.

A distinctive diagnostic feature of CBS is that patients typically maintain insight into the unreality of their hallucinations, distinguishing the condition from psychotic disorders [1]. However, studies indicate that up to 60% of patients initially experience confusion regarding the hallucinatory experiences, particularly when hallucinations appear contextually appropriate [13,19]. The emotional response to CBS hallucinations often differs from that observed in psychiatric disorders. While hallucinations in schizophrenia or bipolar disorder typically elicit distress, CBS hallucinations frequently evoke neutral or positive emotional responses [14,17]. Nevertheless, psychological sequelae may include significant concerns about mental health status. Menon documented that 63% of interviewed patients feared being labeled as mentally ill and 33% were concerned about developing dementia or psychosis due to their hallucinations [1].

Episodes typically last from seconds to minutes, though durations of 1–60 min are most common, with some patients reporting hallucinations lasting hours [17,20]. The frequency can range from rare isolated episodes to multiple daily occurrences. Hallucinations may present in episodic, periodic, or continuous patterns. Episodic hallucinations occur frequently over periods ranging from days to months; periodic hallucinations appear with inconsistent frequency over extended periods; and continuous hallucinations, the least common pattern, occur daily for prolonged durations [17]. Several factors appear to facilitate hallucination onset, including fatigue, somnolence, stress, intense illumination, darkened environments, sensory deprivation, embarrassment, social isolation, and rapid visual deterioration [17]. Notably, the onset of hallucinations often correlates with acute vision loss or fluctuations in visual acuity [15,21]. Conversely, certain interventions may terminate hallucinations once they have commenced: transitioning from poorly illuminated to well-illuminated environments, directly gazing at the hallucination, blinking, or verbally addressing the hallucination, or approaching the hallucinated entity [14,16,22].

Having established the clinical landscape of CBS, its prevalence, risk factors, and phenomenological characteristics, we now turn to the underlying neural mechanisms that may explain these distinctive visual experiences. While the clinical presentation is well characterized, the neurobiological foundations require deeper examination through multiple complementary perspectives.

## 3. Mechanisms of Visual Hallucinations in Charles Bonnet Syndrome

### 3.1. The Deafferentation Hypothesis: Neurophysiological and Neurochemical Considerations

The deafferentation hypothesis constitutes the historical and conceptual cornerstone of pathophysiological accounts of Charles Bonnet syndrome. In its classical formulation, deafferentation refers to the partial or complete loss of afferent sensory input to the visual cortex resulting from peripheral or subcortical damage along the visual pathway [23]. In CBS, this sensory deprivation most commonly arises from ophthalmological conditions such as age-related macular degeneration, glaucoma, diabetic retinopathy, cataracts, or optic nerve pathology, leading to reduced or unreliable retinal input despite preserved cortical integrity [6,14].

At a basic neurobiological level, deafferentation disrupts the normal balance between bottom-up sensory drive and intrinsic cortical activity. Visual cortex, deprived of its primary source of structured input, does not become silent. Instead, converging evidence from animal models and human neurophysiology indicates that sensory deprivation triggers a range of compensatory plastic mechanisms aimed at maintaining baseline levels of activity [23,24]. These include increased spontaneous firing rates, altered synaptic gain, changes in inhibitory interneuron function, and modifications of receptive field properties. Such changes are often described under the umbrella of homeostatic plasticity, whereby neural systems adjust their excitability to stabilize overall firing rates in the face of reduced input. Crucially, deafferentation is neither spatially uniform nor temporally static. Acutely, loss of afferent input weakens feedforward signaling from the retina through the lateral geniculate nucleus to primary visual cortex, reducing the precision and reliability of sensory evidence [8]. Over longer timescales, chronic deprivation induces reorganization across multiple hierarchical levels of the visual system. Functional and metabolic imaging studies in CBS patients reveal altered activity not only in early visual areas but also in extrastriate and higher-order visual regions, including category-selective areas within the ventral stream [25,26]. This suggests that deafferentation propagates through distributed visual networks rather than remaining confined to the primary sensory cortex.

The phenomenological characteristics of CBS hallucinations initially appeared to strongly support deafferentation-based accounts. Hallucinations frequently emerge within regions of visual field loss, and neuroimaging studies demonstrate activation of content-specific cortical areas during hallucinatory episodes, for example, fusiform face area activation during face hallucinations or color area engagement during chromatic percepts [27]. These findings indicate that hallucinations recruit the same neural substrates involved in normal perception, consistent with the idea that internally generated activity within deafferented cortex can give rise to perceptual experience.

However, several empirical and conceptual limitations challenge a purely deafferentation-based explanation. First, sensory deprivation is exceedingly common in aging populations, yet only a minority of visually impaired individuals develop CBS [13,14]. Second, the content of CBS hallucinations is often highly structured, semantically rich, and contextually meaningful, properties that are difficult to reconcile with models based solely on random or noisy spontaneous firing [15]. Third, hallucinations are typically episodic and state-dependent rather than persistent, suggesting dynamic modulation rather than fixed cortical hyperexcitability [20]. These observations support a reinterpretation of deafferentation not as a direct causal mechanism for hallucinations, but as a permissive condition that destabilizes normal perceptual inference [25]. By degrading the reliability and precision of bottom-up sensory signals, deafferentation shifts the balance of perceptual processing toward internally generated activity. In this context, spontaneous or top-down-driven neural activity is less effectively constrained by sensory evidence and may be misinterpreted as externally caused perception.

In summary, the authors of this review consider deafferentation a necessary but insufficient component of CBS pathophysiology. It initiates a cascade of plastic and dynamic changes across the visual hierarchy that render perceptual systems vulnerable to hallucinations, but it does not, on its own, determine when hallucinations occur or what form they take. Understanding CBS, therefore, requires situating deafferentation within a broader network-based and inferential account of visual perception.

While deafferentation explains why the visual system becomes vulnerable to hallucinations, it does not adequately address how these hallucinations acquire their specific content and temporal dynamics. This is where the release phenomenon theory offers a complementary perspective, addressing the mechanistic implementation of how deafferented visual circuits generate structured hallucinatory content.

### 3.2. Release Phenomenon Theory and Computational Evidence

Within Marr’s classical framework [26], theories of Charles Bonnet syndrome can be meaningfully distinguished across computational, algorithmic, and implementational levels. While deafferentation describes a necessary condition at the level of input statistics and circuit perturbation, release phenomena operate primarily at the level of neural implementation. Release phenomena refer to the process where spontaneous neural activity emerges or is ‘released’ when normal inhibition or sensory input is removed. It is similar to when a brake is released, and a previously controlled system becomes independently active. They specify how concrete biological and computational mechanisms transform reduced sensory input into structured internal activity capable of supporting hallucinations [24,28].

Release models explicitly address how visual cortical circuits respond to sustained deprivation through homeostatic plasticity and gain control mechanisms [28,29]. Rather than positing merely an abstract increase in excitability, these models describe specific implementational processes, such as synaptic scaling, altered inhibitory feedback, and changes in recurrent connectivity, that systematically reconfigure network dynamics in response to reduced afferent drive. Synaptic scaling is a self-regulatory mechanism through which neurons adjust the strength of all their synaptic connections to maintain appropriate activity levels. It functions like a cellular thermostat that increases or decreases neural sensitivity to compensate for changes in sensory input. These mechanisms provide a biologically plausible account of how internally generated representations emerge gradually, selectively, and in a spatially organized manner following sensory loss [30,31].

#### 3.2.1. Computational Modeling of Release Phenomena

Computational modeling has provided significant insights into the mechanisms underlying the release phenomenon in CBS. Reichert et al. [32] developed a groundbreaking computational model using a deep Boltzmann machine (DBM) as a generative model of the visual cortex, representing not only the first concrete computational model of CBS but also a pioneering application of DBMs as neuroscience tools to investigate pathological perceptual states. The DBM model successfully reproduced several key clinical features of CBS that had previously lacked a unified explanation, including the initial latency period before hallucination onset, often observed clinically as a delay between vision loss and the appearance of the first hallucinations, as well as the localization of hallucinations to specific areas of visual field damage, reflecting the spatial correspondence between scotomata and hallucination content in patients. According to this framework, hallucinations emerge when homeostatic mechanisms attempt to maintain normal activity levels despite substantially reduced sensory input. Neurons gradually increase their excitability to compensate for sensory deprivation, eventually generating internal representations that are perceived as visual hallucinations. Importantly, the model supports a generative view of cortical function, in which the brain continuously predicts the visual environment and compares these predictions with incoming sensory data, a process that is disrupted in CBS. Overall, this computational approach offers a mechanistic explanation for the systematic progression from visual loss to structured hallucinations, capturing both the temporal dynamics and content specificity characteristic of the clinical syndrome.

#### 3.2.2. Neurochemical Basis of Release Phenomena

The neurochemical underpinnings of release phenomena initially focused on an altered balance between inhibitory (GABA) and excitatory (glutamate) neurotransmission, a seemingly straightforward explanation for hallucination generation [23,24]. However, recent empirical findings have necessitated a more nuanced perspective. Bridge et al. [33] found no consistent differences in GABA+ (GABA plus macromolecules) or glutamate-glutamine complex concentrations between CBS patients and visually impaired controls without hallucinations when measured during non-hallucinatory states. These findings suggest that static alterations in major neurotransmitter systems may not fully explain the release phenomenon. Instead, Bridge et al. [33] proposed that neurochemical changes might be dynamic and transient, occurring primarily during hallucinatory episodes rather than representing permanent alterations in cortical neurochemistry [33]. This perspective aligns with the episodic nature of CBS hallucinations and may explain why consistent neurochemical markers have proven elusive [20,34]. It is important to note that these neurochemical findings from Bridge et al. [33] represent preliminary evidence based on relatively small sample sizes. While they challenge simpler neurochemical explanations, further replication with larger cohorts and more diverse methodologies is needed to establish definitive conclusions about the role of GABA and glutamate in CBS.

Reichert et al.’s [32] computational model proposes a particularly important role for acetylcholine in modulating the release phenomenon. According to their model, acetylcholine functions as a precision regulator, modulating the balance between feedforward and feedback processing at multiple levels of the cortical hierarchy. Reduced cholinergic signaling may favor top-down influences over bottom-up sensory processing, potentially facilitating the generation of hallucinations through enhanced feedback from higher-order visual areas to the early visual cortex. This mechanism is consistent with the well-established role of acetylcholine in attentional modulation and perceptual processing [25,35].

The release phenomenon theory, substantiated by both computational modeling and neurochemical investigations, provides a sophisticated framework for understanding how disruption of normal hierarchical visual processing leads to the complex visual hallucinations characteristic of CBS [8,36]. This model extends significantly beyond simple cortical hyperexcitability to incorporate dynamic interactions between bottom-up and top-down processing streams, as well as homeostatic mechanisms that maintain cortical function despite compromised sensory input. By addressing both the mechanistic “how” and the temporal dynamics of hallucination development, release models represent a crucial advancement in our understanding of CBS pathophysiology [32,37].

Despite its explanatory power, the release phenomenon theory also faces limitations. The computational models, while impressive in reproducing key features of CBS, rely on simplified representations of cortical circuits that may not capture the full complexity of the human visual system. Furthermore, the neurochemical evidence remains somewhat inconclusive, with dynamic rather than static alterations making it difficult to establish consistent biomarkers. Perhaps most importantly, release models primarily address local circuit dynamics but do not fully account for the global brain network interactions that may contribute to the rich phenomenology of CBS hallucinations. The complex content of hallucinations, their relationship to personal and cultural factors, and the preservation of insight all suggest that higher-order cognitive processes beyond basic release mechanisms may be involved in shaping the hallucinatory experience.

The mechanisms discussed thus far, deafferentation and release phenomena, have advanced our understanding of CBS beyond simplistic accounts. However, recent evidence suggests that these explanations, while necessary, remain insufficient to fully explain the phenomenological richness and variability of CBS hallucinations. We now explore emerging perspectives that situate these mechanisms within broader frameworks of neural dynamics and predictive processing.

## 4. Emerging Perspectives: Beyond Simple Hyperexcitability

### 4.1. Neural Desynchronization and Hierarchical Predictive Processing

Neural desynchronization refers to the loss of temporal coordination between different brain areas that normally work together. It is similar to musicians in an orchestra losing their common rhythm, compromising the harmony of the entire performance.

Within a computational framework, hallucinations arise when the precision assigned to sensory prediction errors is systematically reduced, allowing higher-level priors to dominate perceptual inference even in the absence of corroborating input. This perspective fundamentally reframes our understanding of CBS hallucinations, shifting the focus from local hyperexcitability to global disruptions in inferential processing across the visual hierarchy. According to predictive processing frameworks, visual perception emerges from the dynamic interplay between bottom-up sensory signals, top-down predictions, and precision-weighting mechanisms [38,39]. Bottom-up signals convey information from the retina to higher visual areas, carrying prediction errors that indicate discrepancies between expected and actual sensory input. Top–down predictions, generated by higher-order brain regions, constrain the interpretation of sensory information based on prior knowledge and contextual cues. Precision-weighting of prediction errors describes how the brain assigns different levels of importance (or ‘weight’) to errors that arise when brain predictions do not match sensory input. It is similar to how we decide how much to trust information based on its presumed reliability. These precision-weighting mechanisms, in turn, adjust the relative influence of prediction errors and prior expectations, effectively modulating the system’s confidence in sensory evidence. In the context of CBS, severe reductions in sensory input due to visual impairment produce several critical consequences. The ongoing generation of top-down predictions continues in the absence of corrective sensory feedback, allowing internally generated visual content to propagate unchecked. This leads to an altered balance between feedforward and feedback signaling within the visual hierarchy, with feedback influences becoming disproportionately influential. As a result, internally generated visual content emerges that is not effectively constrained by external reality, yet retains the structure and organization of normal visual representations. Concurrently, reduced precision-weighting of sensory prediction errors diminishes the system’s ability to distinguish between externally and internally generated activity.

While the predictive processing framework offers substantial explanatory power for CBS phenomena, we must acknowledge that direct empirical testing of these specific mechanisms in CBS populations remains limited. Much of the current application of predictive processing to CBS relies on extrapolation from computational principles, neuroimaging findings in other perceptual domains, and analogies with other conditions featuring altered perception. Future studies specifically designed to test predictive processing hypotheses in CBS patients, perhaps examining precision-weighting mechanisms directly, will be crucial for validating this theoretical framework.

This computational perspective provides a coherent explanation for several otherwise puzzling features of CBS hallucinations, including their semantic organization, which reflects stored visual representations, their episodic nature, which mirrors fluctuations in precision-weighting, and their content specificity, which aligns with the category-selective organization of the visual system.

### 4.2. Neurochemical Mechanisms and Neurotransmitter Systems

From a predictive processing perspective, neuromodulatory systems such as acetylcholine and serotonin regulate the gain and precision of prediction errors, thereby shaping the balance between bottom-up evidence and top-down expectations rather than directly generating perceptual content. Acetylcholine, in particular, plays a crucial role in signaling the expected precision of sensory input. Under normal conditions, high cholinergic tone enhances the gain of sensory units, effectively increasing the weight assigned to bottom-up information. In CBS, alterations in cholinergic signaling may reduce the precision of sensory evidence, allowing prior expectations to exert greater influence over perceptual content. This mechanism could explain why anticholinergic medications sometimes exacerbate hallucinations, while acetylcholinesterase inhibitors have shown promise in reducing CBS symptoms in some case reports [35]. Other neurotransmitter systems may also contribute to CBS pathophysiology through their effects on predictive processing. GABAergic inhibition shapes the specificity and contrast of visual representations, potentially explaining why benzodiazepines can occasionally reduce hallucination severity. Serotonergic systems modulate the integration of sensory and contextual information, potentially accounting for the reported efficacy of some serotonergic agents in CBS treatment [40].

Importantly, this neurochemical perspective suggests that pharmacological interventions might be most effective when targeted toward restoring the balance between bottom-up and top-down influences rather than simply reducing cortical excitability. This hypothesis could guide more rational approaches to CBS treatment development.

### 4.3. Neurophysiological Evidence

At the computational level, altered functional connectivity reflects a breakdown in hierarchical coordination, impairing the system’s ability to integrate predictions and prediction errors across levels of the visual hierarchy. Recent neurophysiological investigations have provided important insights into these functional alterations associated with CBS.

#### 4.3.1. Electroencephalography (EEG) Findings

DaSilva Morgan et al. [41] conducted a comprehensive EEG study comparing CBS patients with visually impaired controls without hallucinations. Their findings revealed that CBS patients exhibited reduced occipital alpha power and alpha-reactivity, suggesting altered inhibitory control in visual processing regions, alongside increased occipital theta power and elevated theta/alpha ratios, indicative of a shift toward slower oscillatory activity associated with reduced sensory precision. Overall, these patients displayed a pattern of cortical slowing in visual areas, consistent with altered processing dynamics rather than simple hyperexcitation. These results support a desynchronization model in which normal oscillatory coordination between visual processing stages is disrupted. Notably, the neurophysiological changes were more pronounced in patients experiencing complex hallucinations compared to those with simple hallucinations, pointing to a potential neural signature associated with hallucination complexity. Piarulli et al. [34] used high-density EEG to investigate dynamic changes in brain activity during active hallucinations in a single case. Their analysis revealed reduced delta and theta power in frontal regions, suggesting alterations in top-down control, while alpha power increased in occipital and posterior medial regions, potentially reflecting enhanced internal generation of visual content. Additionally, small-world properties in theta networks were disrupted, indicating less efficient information transfer, and alpha signal complexity increased in medial frontal, left posterior, and right centroposterior regions, suggesting more chaotic processing dynamics.

Taken together, these EEG findings provide direct evidence that hallucinations in CBS involve complex alterations in neural dynamics and network coordination rather than simple increases in excitability. The observed changes in oscillatory patterns are consistent with predictive processing accounts of CBS, reflecting a disrupted balance between bottom-up and top-down processing within the visual system.

#### 4.3.2. Transcranial Magnetic Stimulation (TMS) Evidence

TMS studies have provided direct evidence regarding cortical excitability in CBS. DaSilva Morgan et al. [41] used phosphene induction to probe visual cortex excitability and found greater variability in phosphene thresholds among CBS patients compared to controls, suggesting unstable excitability rather than consistent hyperexcitability. Moreover, phosphene thresholds were significantly negatively correlated with hallucination severity, indicating that more severe hallucinations were associated with greater instability. Patients experiencing complex hallucinations also exhibited more widespread phosphene induction, reflecting altered spatial specificity of visual cortical responses. These findings imply that although overall phosphene thresholds may not differ markedly between CBS patients and controls, the stability of cortical excitability is compromised in CBS, with increased instability corresponding to more severe hallucinations. This pattern aligns with the predictive processing perspective, which attributes hallucinations to disrupted precision-weighting rather than to a simple increase in excitability.

Beyond its mechanistic insights, the TMS evidence underscores the potential of non-invasive brain stimulation as both a research tool and a therapeutic approach for CBS. By transiently modulating cortical excitability, TMS may help normalize visual processing patterns and potentially reduce the frequency or intensity of hallucinations.

### 4.4. Neuroimaging Evidence

Functional MRI studies have revealed altered patterns of visual cortical activation in CBS that go beyond simple hyperexcitability. In a seminal investigation, Ffytche et al. [27] demonstrated content-specific activation during hallucinations, with specialized visual areas engaging in accordance with the content being hallucinated, for example, face-selective regions activating during face hallucinations. This finding confirmed that hallucinations recruit the same neural substrates involved in normal perception of the corresponding stimuli. More recently, DaSilva Morgan et al. [41] reported reduced activation in primary visual cortex and ventral extrastriate areas in response to visual stimulation among CBS patients, suggesting a paradoxical decrease in responsiveness to external input despite spontaneous internal activation. Similarly, Bridge et al. [33] observed subtle differences in visual responses to object stimuli, with a tendency toward greater activation contrasts between objects and scrambled stimuli, indicating altered categorical processing in the ventral visual stream rather than uniform hyperactivity. Collectively, these findings suggest that the visual cortex in CBS does not merely exhibit increased activity but demonstrates altered activation patterns that reflect disrupted processing hierarchies and imbalances between different visual pathways. The content-specific activation observed during hallucinations provides particularly compelling evidence that hallucinations arise from structured internal representations rather than from random neural firing.

Resting-state functional connectivity analyses have further illuminated network-level alterations underlying CBS hallucinations. Unlike task-based functional magnetic resonance imaging (fMRI) studies, which focus on activation patterns during specific activities, resting-state analyses reveal intrinsic communication patterns between brain regions, offering insights into how hallucinations may emerge from altered network dynamics. Bridge et al. [33] found subtle but significant differences in connectivity between the lateral occipital cortex, critical for object recognition and other brain regions, with a trend toward stronger local connectivity within visual processing areas in CBS patients who experienced more frequent or intense hallucinations. This heightened local connectivity may reflect an internally focused processing loop less constrained by external input, facilitating the emergence of hallucination-like percepts. Complementing these results, Piarulli et al. [34] used high-density EEG to capture connectivity dynamics during active hallucinatory episodes in a case study. They observed altered functional relationships between visual processing regions and components of the default mode network (DMN), a system typically associated with internally directed cognition, autobiographical memory, and self-referential processing. Such changes suggest a mechanism whereby visual representations, normally constrained by the DMN during rest, become inappropriately activated and interpreted as external percepts. Altogether, these findings indicate a fundamental reorganization of information flow within and between visual and non-visual networks in CBS, showing that hallucinations involve altered patterns of communication across distributed neural systems rather than isolated hyperactivity. This network perspective helps explain both the structured content of hallucinations, which draw on existing visual representations and their percept-like phenomenology, arising from altered integration between perceptual and reality-monitoring networks.

In contrast, structural neuroimaging studies have produced inconsistent evidence regarding anatomical differences in CBS patients, offering important clues about the syndrome’s nature. Firbank et al. [42] conducted a comprehensive morphometric analysis comparing CBS patients with visually impaired controls without hallucinations, employing voxel-based morphometry, cortical thickness analysis, and diffusion tensor imaging. They found no significant structural differences after controlling for age, sex, and degree of visual impairment, suggesting that CBS hallucinations may not rely on detectable macroscopic abnormalities beyond those associated with vision loss. Martial et al. [43] reported altered cortical thickness in visual processing regions in a single-case study. However, these findings must be interpreted cautiously due to the lack of age-matched controls and the inherent limitations of single-case designs, which may reflect individual variability rather than hallucination-specific changes. The relative absence of consistent structural alterations in CBS contrasts sharply with conditions such as schizophrenia or dementia with Lewy bodies, where hallucinations typically coincide with detectable brain changes. This dissociation between functional and structural findings supports the view that CBS hallucinations emerge primarily from altered functional dynamics within anatomically preserved circuits rather than from structural damage to specific regions. This perspective aligns with the integrated model developed throughout this review, positing that hallucinations in CBS arise from adaptive responses to sensory deprivation that reorganize functional information processing while preserving structural integrity, with implications for conceptualizing CBS as a functional disorder and for developing interventions targeting neural dynamics rather than structural abnormalities.

These emerging perspectives on neural desynchronization, neurochemical mechanisms, and hierarchical processing offer valuable insights into specific aspects of CBS. The neurophysiological evidence reveals complex patterns of altered oscillatory activity and unstable cortical excitability rather than simple hyperactivation. Neurochemical findings suggest dynamic rather than static alterations in neurotransmitter systems. Neuroimaging studies demonstrate altered functional connectivity patterns across distributed networks rather than isolated cortical changes. Collectively, these findings point to CBS as a network-level disorder involving disrupted coordination across multiple brain systems. However, a comprehensive understanding requires an integrated model that synthesizes these diverse perspectives across multiple levels of explanation, from cellular adaptations to network dynamics to computational principles. The following section presents such a model, drawing together the empirical and theoretical threads explored thus far into a unified framework that bridges these complementary levels of analysis.

## 5. Integrated Model: Neural Desynchronization and Selective Disinhibition

The body of evidence accumulated over the past several decades has gradually shifted scientific understanding of CBS from earlier models centered solely on cortical hyperexcitability toward a more nuanced, integrated perspective. Classic accounts provided valuable initial frameworks, but recent advances across multiple disciplines now enable a more comprehensive synthesis. As we have seen, deafferentation provides the initial condition necessary for CBS but cannot explain the structured content or episodic nature of hallucinations. Release phenomena help explain how deafferented cortex generates structured visual content, but struggle to account for the global network dynamics involved. The neurophysiological evidence reveals complex patterns of altered oscillatory activity and unstable excitability rather than simple hyperactivation, while neuroimaging findings demonstrate altered functional connectivity across distributed networks rather than isolated regional changes. These observations, together with insights from predictive processing frameworks (Section 4.1) and neurochemical mechanisms, demand a more sophisticated explanatory model.

By synthesizing these diverse insights from computational neuroscience, neurophysiology, neuroimaging, and clinical observations, we propose a model of CBS pathophysiology that operates across multiple levels of analysis and explanation, building upon earlier network-based approaches [8,26]. This integrated model centers on neural desynchronization and selective disinhibition as key mechanisms that link the various empirical findings and theoretical perspectives discussed thus far.

### 5.1. A Multilevel Explanatory Framework

The preceding sections have addressed Charles Bonnet syndrome at complementary levels of explanation, each capturing essential but partial aspects of its pathophysiology. Deafferentation describes the altered sensory input regime imposed by visual impairment, release phenomena specify the neural mechanisms through which cortical systems respond to sustained deprivation, and predictive processing frameworks formalize the inferential principles governing perceptual experience under uncertainty. The integration of these perspectives provides a unified, multilevel model of CBS grounded in Marr’s framework of computational neuroscience [26].

**Figure 2:**
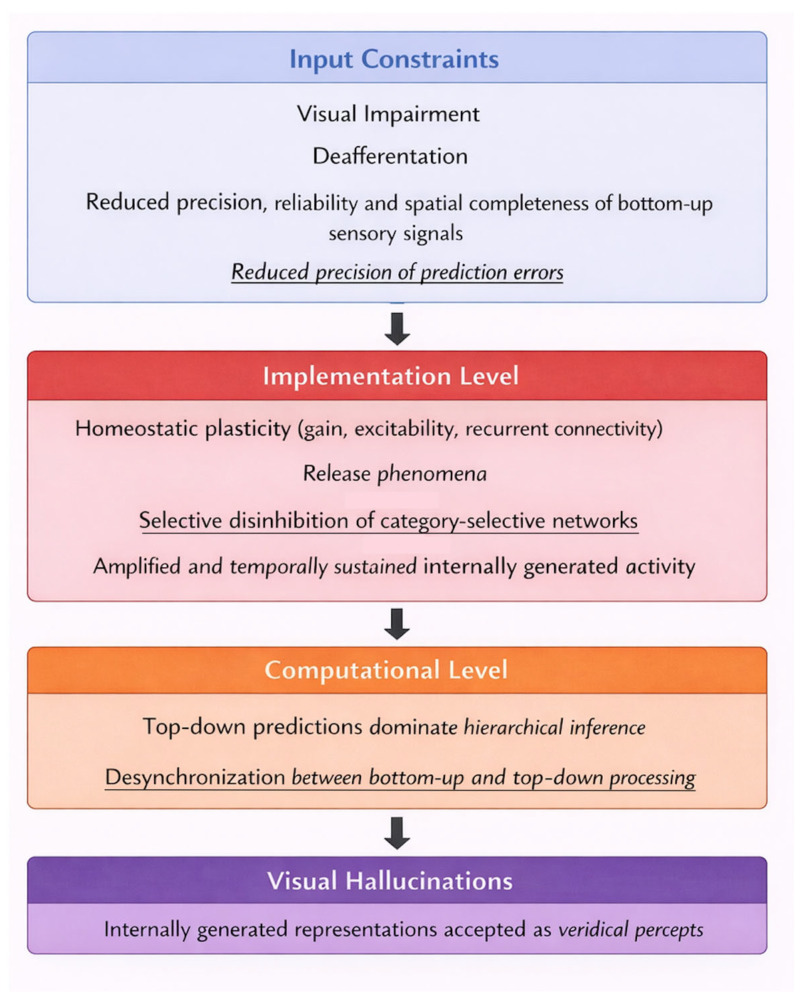
Schematic representation of how hallucinations arise following visual impairment according to the proposed multilevel explanatory framework.

At the level of input constraints, visual deafferentation reduces the precision, reliability, and spatial completeness of bottom-up sensory signals. This alteration does not merely weaken sensory drive but fundamentally changes the statistical structure of the perceptual environment encountered by the visual system. As a result, perceptual inference must operate under conditions of chronically elevated uncertainty, particularly in deafferented regions of the visual field. At the implementation level, neural systems adapt to this altered input regime through homeostatic and plastic mechanisms that regulate gain, excitability, and recurrent connectivity. Release phenomena emerge as a consequence of these adaptations, enabling internally generated activity within higher-order visual areas to become amplified and temporally sustained. Importantly, the activity that is released reflects learned representational structure and semantic organization rather than undifferentiated noise, providing a mechanistic substrate for the content-rich nature of CBS hallucinations, as demonstrated in computational simulations [32]. At the computational level, predictive processing models explain how these implementation-level changes translate into conscious perceptual experience [38,39]. Reduced sensory precision attenuates the impact of prediction errors, allowing top-down predictions to dominate hierarchical inference. Under these conditions, internally generated representations are more likely to be accepted as veridical percepts, particularly when hierarchical coordination and temporal synchronization between visual areas are disrupted. Hallucinations thus arise not from a failure of perception per se, but from a systematic shift in the inferential balance that normally distinguishes internally generated activity from externally caused sensory input [44], as schematized in Figure 2.

### 5.2. Key Components of the Integrated Model

According to this integrated perspective, several interconnected mechanisms contribute to CBS hallucinations. Deafferentation and subsequent cellular adaptation play a central role: loss of visual input triggers homeostatic processes that alter the excitability of deafferented neurons. Rather than resulting in uniform hyperexcitability, these adaptations produce complex patterns of altered neural dynamics, including receptor upregulation, changes in local inhibitory circuit function, and modifications in synaptic strength, which collectively reshape the response properties of visual neurons. This view is supported by recent evidence of variable rather than uniformly decreased phosphene thresholds in CBS patients [37]. Disrupted hierarchical processing further contributes to hallucinations, as the visual system normally relies on a balance between bottom-up sensory input and top-down predictions. In CBS, diminished bottom-up signals allow unconstrained top-down influences to dominate perception, a phenomenon reflected in fMRI findings showing that hallucinations recruit the same category-selective regions involved in normal perception of corresponding stimuli [27]. At the network level, CBS is characterized by altered synchronization between distributed brain networks rather than isolated cortical hyperactivity. EEG studies reveal changes in oscillatory patterns that provide direct evidence of desynchronization, particularly between early visual areas and higher-order regions involved in object recognition, attention, and semantic processing, which may compromise the brain’s ability to distinguish internally generated activity from external sensory input [34]. This is complemented by selective disinhibition, in which specific neural circuits become disinhibited based on their pre-existing organization and connectivity patterns. Such selectivity explains why hallucinations often contain semantically meaningful and structured content, frequently reflecting culturally familiar objects and faces rather than random visual features, as observed by Ffytche in his investigations of content-specific hallucination mechanisms [8]. Finally, the transient, state-dependent nature of hallucinations suggests that these neural alterations interact with fluctuating brain states influenced by factors such as arousal, attention, and environmental context. This state dependence accounts for the episodic occurrence of hallucinations and their modulation by changes in lighting, attention, or general arousal level, patterns that cannot be easily reconciled with models based solely on hyperexcitability.

It is important to distinguish between components of this model that have strong empirical support and those that remain more hypothetical. The role of deafferentation as an initial trigger and the content-specific activation during hallucinations are well-established empirically. However, specific claims about the role of neurotransmitters in precision-weighting and the exact nature of desynchronization between hierarchical levels represent theoretical extensions that, while consistent with available evidence, require further direct investigation in CBS populations.

Our perspective could extend and complement the Perception and Attention Deficit (PAD) model proposed by Collerton et al. [25], which emphasizes the interaction between impaired sensory processing and top-down attentional mechanisms in generating complex visual hallucinations. While the PAD model focuses primarily on the functional interplay between attentional binding and object perception within scene representations, our framework adds a crucial mechanistic layer by specifying the neurophysiological substrate underlying these interactions: neural desynchronization. Where Collerton et al. [25] highlight the behavioral and cognitive consequences of combined attentional and perceptual deficits, our model illuminates the dynamic neural processes through which these deficits manifest, specifically, the disruption of synchronized activity between distributed visual networks. This desynchronization mechanism provides a neurophysiological explanation for how the attentional and perceptual components identified in the PAD model interact at the implementation level.

Our approach also builds upon the comprehensive review by Christoph et al. [45], who similarly highlight deafferentation, predictive coding, and the PAD model as key mechanisms in CBS pathophysiology. While their analysis provides an excellent synthesis of existing theoretical frameworks, our focus on desynchronization as a unifying neurophysiological principle offers a novel perspective that integrates these diverse accounts. Where Christoph et al. [45] present these mechanisms as parallel explanatory frameworks, our model proposes that neural desynchronization represents a common pathway through which various pathophysiological processes ultimately manifest as hallucinations.

### 5.3. Explanatory Power of the Integrated Model

This integrated perspective provides an explanatory framework for key observations that are not adequately accounted for by simple hyperexcitability models. It accounts for the semantic richness and organization of hallucination content, which reflects pre-existing representational structures rather than random neural firing [32], as well as the episodic nature of hallucinations, consistent with fluctuations in network states rather than continuous hyperactivity. The model also explains the lack of correlation between the severity of visual impairment and hallucination complexity, indicating that factors beyond deafferentation alone shape the characteristics of hallucinations.

This integrated model also reconciles several apparently contradictory findings in the CBS literature. First, it resolves the paradox of why CBS patients show both increased and decreased cortical activity in different studies. Our model explains this through region-specific alterations in excitation-inhibition balance rather than global hyperexcitability. Second, it addresses the contradiction between preserved insight and vivid hallucinations by distinguishing between perceptual inference mechanisms (which are disrupted) and higher-order reality monitoring systems (which remain intact). Third, it explains the puzzling observation that improving visual input sometimes worsens hallucinations before improving them, a finding consistent with temporary destabilization of predictive processing as the system recalibrates to new precision weightings. Fourth, it reconciles the variable efficacy of pharmacological interventions targeting different neurotransmitter systems, as these may address different components of the desynchronized network depending on individual patient characteristics. Finally, it explains why hallucination content often reflects culturally familiar imagery despite arising from deafferentation. The released activity draws upon existing representational structures shaped by prior experience rather than random neural firing.

Neurophysiological evidence further supports this view, revealing complex patterns of altered cortical activity and connectivity rather than straightforward excitatory changes [37,41]. Additionally, the preservation of insight in most CBS patients suggests that reality-monitoring systems remain at least partially intact despite altered perceptual processing, a finding consistent with predictive processing accounts that distinguish between perceptual inference and higher-order belief evaluation [38,44]. The variable efficacy of different treatment approaches underscores the likelihood that hallucinations arise from multiple interacting mechanisms rather than a single pathophysiological process. By conceptualizing CBS as a disorder of neural synchronization and predictive inference, this integrated model offers a more comprehensive framework for understanding the syndrome’s diverse manifestations and for developing targeted therapeutic strategies, extending earlier network-based models to incorporate advances in computational neuroscience and predictive processing theory [8,25].

While building on the foundations established by previous frameworks, such as the PAD model, and synthesized in recent reviews, such as that of Christoph et al. [45], our desynchronization-centered approach adds mechanistic specificity that may guide future research and therapeutic interventions. By focusing on the temporal dynamics of neural activity rather than simply its amplitude or location, this model opens new avenues for investigation using techniques that can directly assess neural synchronization, such as magnetoencephalography (MEG), high-density EEG, and connectivity analyses of functional neuroimaging data.

The integrated neural desynchronization model presented above has significant implications beyond theoretical understanding. By reconceptualizing CBS as a disorder involving complex interactions across multiple neural systems rather than simple hyperexcitability, this framework opens new avenues for diagnosis and treatment that more precisely target the underlying mechanisms.

To clarify the added value of our framework and to explicitly contrast it with previous accounts, Table 1 summarizes the principal theoretical models of Charles Bonnet syndrome across levels of explanation, core mechanisms, and explanatory scope. It highlights the specific contributions of neural desynchronization, selective disinhibition, and precision-weighting within a multilevel framework.

### 5.4. Empirical Predictions and Testable Hypotheses

To operationalize this framework and provide concrete guidance for future empirical studies, we formulate several specific, testable hypotheses that follow directly from the proposed integration of deafferentation, hierarchical inference, and neural desynchronization. These predictions span multiple levels of analysis and can be addressed using currently available neurophysiological and neuroimaging techniques.

At the level of oscillatory dynamics, we predict that the phenomenological distinction between simple and complex hallucinations will be reflected in distinct patterns of neural activity. Specifically, simple geometric hallucinations, such as grids, zigzags, or colored patches, should be associated with increased alpha and beta power (8–30 Hz) localized to early visual cortex (V1/V2), reflecting altered local gain regulation and intrinsic excitability. In contrast, complex semantic hallucinations involving faces, objects, or scenes should recruit category-selective regions in the ventral visual stream and be characterized by increased gamma-band activity (30–80 Hz), which is known to support feature binding and semantic processing. These oscillatory signatures can be directly tested using source-localized high-density EEG or MEG recordings during active hallucination episodes.

From the perspective of hierarchical connectivity, our model predicts systematic alterations in the balance between bottom-up and top-down information flow during hallucinatory experiences. Specifically, patients should exhibit reduced effective connectivity from early visual cortex to higher-order visual areas, reflecting weakened sensory constraints on perceptual inference, alongside increased top-down connectivity from frontal and parietal regions to visual cortex, reflecting the dominance of prior expectations. These directional connectivity patterns can be quantified through dynamic causal modeling or Granger causality analysis applied to fMRI or EEG data, providing a direct test of the hierarchical imbalance central to predictive processing accounts of hallucinations.

At the network level, we hypothesize that the episodic nature of CBS hallucinations reflects transient disruptions in large-scale functional integration. Hallucinatory episodes should coincide with reductions in functional connectivity, measured via coherence, phase-locking, or other synchronization metrics, between early visual cortex and frontoparietal control networks. This desynchronization would compromise the brain’s ability to effectively distinguish internally generated activity from externally driven sensory input. Time-resolved connectivity analysis comparing periods with and without hallucinations within the same patients would provide a powerful approach to testing this prediction, controlling for individual differences and capturing the dynamic nature of the phenomenon. Individual differences in hallucination phenomenology may also be systematically related to baseline neural architecture. We predict that the specific content of hallucinations, for example, whether patients predominantly experience faces, objects, or scenes, will correlate with the strength of resting-state functional connectivity between early visual cortex and corresponding category-selective regions in the ventral stream. For instance, patients with stronger connectivity between V1 and the fusiform face area may be more likely to experience face hallucinations. This prediction can be tested through correlation analyses relating baseline connectivity profiles to detailed phenomenological characterization of hallucination content.

Finally, our framework generates predictions regarding treatment response that may have direct clinical utility. We hypothesize that effective therapeutic interventions, whether neuromodulatory (e.g., transcranial direct current stimulation) or pharmacological (e.g., cholinergic agents), will restore more balanced hierarchical coordination and reduce pathological desynchronization between visual and control networks. These neural changes should be measurable as alterations in effective connectivity and oscillatory coupling patterns from pre- to post-treatment assessments, and their magnitude may correlate with clinical improvement in hallucination frequency or severity. Collectively, these falsifiable predictions provide concrete targets for future investigation using high-density EEG and MEG, multimodal neuroimaging approaches combining fMRI with electrophysiology, and longitudinal study designs incorporating real-time phenomenological reporting. By translating the present theoretical framework into specific empirical hypotheses, we aim to facilitate the transition from conceptual models to data-driven refinement of our understanding of Charles Bonnet Syndrome pathophysiology.

### 5.5. Multifactorial Influences and Integrated Care

While this review focuses primarily on neural mechanisms, it is important to acknowledge that CBS is a multifactorial syndrome influenced by factors beyond neural circuitry. Psychological, peripheral visual system, and environmental factors all contribute to the manifestation and experience of visual hallucinations in visually impaired individuals.

Psychological factors, including stress, anxiety, and social isolation, have been identified as potential modulators of hallucination experiences [7,12,17]. From the perspective of our integrated model, these psychological states may influence hallucination threshold and content by affecting the precision-weighting mechanisms central to predictive processing. Stress and anxiety can alter arousal levels and neurotransmitter balance, potentially destabilizing the already compromised balance between bottom-up and top-down processing in visually impaired individuals. Social isolation may reduce external sensory stimulation and contextual anchoring, further enabling internally generated content to dominate perception. Conversely, social engagement and psychological well-being may strengthen reality-monitoring processes and provide competing sensory input that constrains hallucinatory experiences.

Peripheral visual system factors also play important roles beyond simply initiating deafferentation. The specific pattern of visual loss (central vs. peripheral, gradual vs. sudden, complete vs. partial) may influence both the likelihood of developing hallucinations and their phenomenological characteristics [22]. For instance, the spatial correspondence between scotomata and hallucination content suggests that the precise topography of retinal damage shapes hallucinatory experiences. Additionally, fluctuations in intraocular pressure, retinal blood flow, or medication effects on the peripheral visual system may contribute to the episodic nature of hallucinations by dynamically altering the quality and quantity of residual visual input.

Environmental conditions, including lighting, visual complexity, and multisensory stimulation, can significantly modulate hallucination frequency [14,17]. These factors likely operate by altering the reliability and precision of available sensory evidence, thereby shifting the balance of predictive processing. Improved lighting may enhance the precision of residual visual input, allowing bottom-up signals to more effectively constrain perceptual inference. Similarly, multisensory stimulation may provide alternative sources of reliable sensory evidence that reduce reliance on vision-based predictions.

Our neural desynchronization model provides a framework for understanding how these diverse factors converge to influence hallucination experiences. By conceptualizing hallucinations as arising from altered precision-weighting and network synchronization, we can explain how psychological, peripheral, and environmental factors modulate CBS symptoms through their effects on these core neural mechanisms. This perspective suggests that comprehensive clinical care should address multiple contributing factors rather than focusing exclusively on neural or ophthalmological interventions. An integrated approach combining visual rehabilitation, psychological support, environmental modifications, and, when appropriate, pharmacological or neuromodulatory treatments, is likely to be most effective for managing this complex syndrome. To illustrate the theoretical clinical implications derived from each explanatory framework, Table 2 summarizes the major theoretical models of CBS alongside their corresponding therapeutic perspectives.

## 6. Clinical Implications and Treatment Approaches

### 6.1. Diagnostic Considerations

The integrated model of CBS pathophysiology outlined in this review has important implications for clinical diagnosis and patient assessment. Current diagnostic practices largely emphasize the exclusion of psychiatric or neurocognitive disorders in visually impaired individuals who report hallucinations. However, the marked heterogeneity in clinical presentation and the growing evidence for multiple interacting neural mechanisms suggest that CBS is better conceptualized as a spectrum of related conditions rather than a single, uniform entity [9]. Viewing CBS as a network-level disorder characterized by disruptions across multiple levels of processing calls for a refinement of existing diagnostic approaches. In particular, clinical assessment would benefit from a more detailed phenomenological characterization of hallucinations, encompassing both simple and complex forms, as well as their content, frequency, temporal dynamics, and contextual triggers. Such comprehensive profiling may help identify distinct neurobiological subtypes that differ in underlying mechanisms and, consequently, in their responsiveness to specific interventions. This reconceptualization also has implications for the assessment of insight, traditionally considered a defining feature of CBS. While insight is often preserved, its degree may fluctuate over time or across episodes, and early hallucinatory experiences can be accompanied by confusion or distress before insight is fully established. Diagnostic tools should therefore accommodate graded levels of insight and capture patients’ emotional responses, rather than treating insight as a binary criterion. Moreover, the neurophysiological and neuroimaging evidence reviewed here points to the potential development of objective biomarkers to complement subjective clinical reports. Measures derived from functional connectivity analyses, oscillatory dynamics, or indices of sensory precision could, in the future, aid in differentiating CBS from other causes of visual hallucinations and in tracking disease evolution. Equally important is the systematic evaluation of comorbid factors, including cognitive status and mood. Interactions between visual impairment, affective symptoms, and cognitive function appear to influence both the expression of hallucinations and treatment response, with recent findings indicating that depression and anxiety may modulate hallucination severity even in younger CBS patients [46].

Finally, the episodic and dynamic nature of CBS hallucinations underscores the importance of longitudinal monitoring. Reliance on cross-sectional assessments alone may obscure meaningful changes in hallucination phenomenology over time, whereas longitudinal approaches can provide insights into underlying mechanisms and adaptive processes associated with sensory deprivation. Incorporating these considerations into clinical practice would allow for a more nuanced and precise characterization of CBS, supporting the development of personalized diagnostic and therapeutic strategies. Such an approach is fully consistent with the multilevel explanatory framework advanced in this review, which emphasizes that CBS arises from complex interactions among sensory loss, neural dynamics, and predictive inference rather than from a single pathological mechanism.

### 6.2. Non-Pharmacological Interventions

The theoretical framework developed in this review provides a mechanistic rationale for several non-pharmacological interventions that have demonstrated efficacy in CBS management. By targeting different components of the proposed pathophysiological model, these approaches offer complementary strategies for reducing hallucination frequency and associated distress.

#### 6.2.1. Education and Psychological Approaches

Patient education and reassurance represent perhaps the most consistently effective intervention for CBS. Explaining the benign nature of hallucinations and their relationship to visual impairment significantly reduces associated anxiety and improves quality of life. Cox and Ffytche documented that 94% of patients reported increased comfort following proper explanation of their condition [47]. From the perspective of predictive processing, education may help patients establish appropriate prior expectations that recalibrate the inferential processes underlying hallucination generation. When patients understand the nature of their experiences, top-down knowledge can modulate the interpretation of unusual perceptual events, reducing their disruptive impact. The normalization of experiences through patient support groups or shared testimonials can further mitigate psychological distress. Jones et al. [7] found that many patients benefited from learning about others’ experiences with CBS, which reduced feelings of isolation and fear of mental illness. These psychological interventions address the secondary impact of hallucinations rather than their primary generation, but may indirectly influence hallucination frequency through effects on attention, stress, and arousal, factors known to modulate CBS severity.

#### 6.2.2. Sensory and Environmental Approaches

Visual rehabilitation aims to optimize remaining visual function through appropriate corrective lenses, low-vision aids, or treatment of underlying ocular pathology. These interventions can reduce hallucination frequency by partially restoring reliable bottom-up sensory input, thereby constraining aberrant predictive processes and increasing the precision of sensory evidence relative to prior expectations [6,48]. Even modest improvements in visual acuity or contrast sensitivity may be sufficient to shift the balance of perceptual inference away from hallucination-prone states. Environmental modifications represent practical strategies for managing hallucinations in daily life. Improved lighting conditions, reduced visual clutter, and increased multisensory stimulation may help reduce hallucination frequency by enhancing the reliability of available sensory input and providing stronger constraints on perceptual inference [14]. The efficacy of these approaches supports the view that CBS hallucinations arise from imbalances between sensory evidence and prior expectations rather than irreversible neural damage.

#### 6.2.3. Behavioral Coping Strategies

Furthermore, patients with CBS often develop behavioral coping strategies through experience. These include intentional eye movements, blinking, focusing attention elsewhere, verbally addressing the hallucination, or increasing environmental light levels when hallucinations occur [7]. These techniques may work through several mechanisms: redirecting attention, providing competing sensory input, or actively engaging reality-monitoring processes that help distinguish internally generated content from external perception.

#### 6.2.4. Neuromodulatory Techniques

DaSilva Morgan et al. [49] conducted a randomized, placebo-controlled crossover trial of transcranial direct current stimulation (tDCS) applied to the occipital cortex. They found that anodal (excitatory) stimulation significantly reduced hallucination frequency compared to sham stimulation, with effects persisting for several weeks after the intervention. This finding directly supports the involvement of altered cortical excitability in CBS and demonstrates the potential for targeted neuromodulation to restore more normal patterns of neural activity. An approach that might be tested in the future is to investigate whether training protocols designed to enhance visual processing in remaining functional vision can strengthen bottom-up constraints on perceptual inference. While evidence for these approaches remains insufficient, they represent theoretically grounded interventions consistent with the network-level model of CBS presented in this review.

### 6.3. Pharmacological Approaches

Despite the absence of large-scale controlled trials, several medication classes have shown promise in treating CBS hallucinations. The heterogeneity in treatment responses aligns with the multifaceted pathophysiological model proposed in this review, suggesting that different pharmacological agents may target distinct aspects of the underlying mechanisms.

#### 6.3.1. Modulating Neurotransmitter Systems

Antipsychotic medications, particularly atypical antipsychotics such as olanzapine and quetiapine, have been reported to reduce hallucinations in some CBS patients [50]. These agents primarily modulate dopaminergic signaling, but also affect serotonergic, histaminergic, and other neurotransmitter systems. From a predictive processing perspective, antipsychotics may normalize the precision weighting of sensory evidence relative to prior expectations, potentially restoring a more appropriate balance between bottom-up and top-down influences. However, their use requires careful consideration of potential side effects, particularly in elderly patients who constitute the majority of the CBS population.

Acetylcholinesterase inhibitors such as donepezil and rivastigmine have shown efficacy in case reports and small series [35]. These medications increase cholinergic tone, which plays a crucial role in attentional modulation and the signaling of sensory precision. Enhanced cholinergic signaling may increase the relative weight assigned to bottom-up sensory information, potentially counteracting the dominance of top-down influences that contribute to hallucination generation in CBS. The efficacy of these agents supports the involvement of cholinergic mechanisms in the pathophysiology of CBS and aligns with computational models of hallucination generation that emphasize the role of acetylcholine in balancing feedforward and feedback processing.

Selective serotonin reuptake inhibitors (SSRIs) and serotonin-norepinephrine reuptake inhibitors (SNRIs) have demonstrated benefits in some cases [40]. Serotonergic systems modulate sensory processing, attention, and mood, potentially influencing multiple aspects of the hallucination generation process. The efficacy of these agents suggests a role for serotonergic mechanisms in CBS, though whether their effects are direct (altering visual processing) or indirect (reducing anxiety that may exacerbate hallucinations) remains unclear.

#### 6.3.2. Targeting Neural Excitability

Anticonvulsant medications represent another therapeutic approach for CBS. By stabilizing neural membrane excitability through effects on voltage-gated ion channels or enhanced GABAergic inhibition, these agents may counteract the altered excitation-inhibition balance proposed to contribute to CBS hallucinations. Case reports suggest efficacy for medications such as gabapentin, carbamazepine, and valproate [51]. These findings align with the concept that unstable or dysregulated neural activity, rather than simple hyperexcitability, contributes to CBS pathophysiology.

One might hypothesize that benzodiazepines could reduce CBS hallucinations through enhancement of GABA-mediated inhibition, particularly in individuals with elevated visual cortex excitability. However, evidence for their effectiveness in CBS remains lacking, with early observational studies reporting no benefit for hallucination frequency [13,14]. Additionally, their sedating properties and risk of cognitive side effects limit their utility, particularly in elderly patients who may be more susceptible to adverse effects, including falls and confusion.

#### 6.3.3. Rational Pharmacotherapy Based on Pathophysiological Mechanisms

The variety of medication classes that have shown benefit in CBS supports the integrated model presented in this review, suggesting that hallucinations may arise through multiple interacting mechanisms rather than a single pathophysiological process. This heterogeneity highlights the potential value of personalizing pharmacological approaches based on individual patient characteristics and hallucination phenomenology. For example, patients whose hallucinations appear to involve primarily increased excitability (e.g., simple, geometric hallucinations) might respond better to anticonvulsants, while those with complex, semantically rich hallucinations suggesting top-down influences might benefit more from cholinergic enhancement. Similarly, the presence of comorbid anxiety or depression might favor serotonergic agents that address both hallucinations and mood symptoms. While such personalized approaches remain speculative pending larger controlled trials, they represent a rational extension of the multilevel explanatory framework developed throughout this review. Future research that correlates treatment response with specific neurophysiological or phenomenological markers could substantially advance our ability to target pharmacological interventions to underlying mechanisms.

The diversity of reported treatment responses is consistent with the multilevel pathophysiological framework proposed in this review, but the empirical support for specific interventions remains limited and uneven. Overall, the available literature does not support the existence of an evidence-based, standardized treatment protocol specific to CBS. Current pharmacological approaches remain empirical and mechanism-informed rather than trial-validated. Treatment decisions should therefore be individualized, carefully weighing potential benefits against risks, particularly in elderly patients. In light of the methodological variability and limited strength of the available data, Table 3 provides a structured summary of therapeutic interventions in CBS, including their study design, level of evidence, and key limitations. This overview makes explicit the predominantly very low level of evidence currently supporting most interventions, with all pharmacological treatments based exclusively on case reports or small uncontrolled case series.

## 7. Future Research Directions

The integrated model of CBS presented in this review suggests several promising avenues for future investigation. By approaching CBS as a complex disorder involving interactions across multiple levels, from cellular mechanisms to network dynamics to inferential processing, researchers can develop more comprehensive and targeted approaches to understanding and treating this condition.

### 7.1. Neurodynamic Investigations

A major limitation of the current literature is the relative scarcity of data capturing neural activity during ongoing hallucinatory episodes. Addressing this gap represents a critical priority for future research, as real-time monitoring of brain activity during hallucinations has the potential to substantially advance our understanding of CBS. The integration of high-density EEG or magnetoencephalography (MEG) with experience-sampling methodologies would make it possible to characterize the neural dynamics that precede, accompany, and follow hallucinatory experiences with high temporal resolution. Such approaches could help determine whether specific neural signatures reliably predict hallucination onset and whether these signatures vary systematically as a function of hallucination content or complexity. Within this context, further investigation of oscillatory dynamics constitutes a particularly promising avenue. More detailed characterization of frequency-specific oscillatory patterns and their relationship to hallucination phenomenology could shed light on the mechanisms underlying different hallucinatory experiences. While our integrated model generates specific predictions regarding distinct oscillatory profiles for simple versus complex hallucinations (Section 5.4), empirical validation of these predictions remains a priority. Similarly, determining whether effective therapeutic interventions selectively normalize specific aspects of aberrant oscillatory activity represents a critical test of mechanism-based treatment approaches. Addressing these issues would provide direct empirical tests of the neural dynamics proposed by the integrated model outlined in this review.

In addition, the study of cross-frequency coupling, such as interactions between theta and gamma bands, may offer crucial insights into how disrupted hierarchical processing contributes to hallucinations in CBS. Under normal conditions, cross-frequency coupling supports the integration of information across multiple temporal and spatial scales in perceptual processing. Alterations in these coupling patterns could impair coordination between different levels of the visual hierarchy, providing a mechanistic account of how internally generated activity comes to be misattributed to external sensory sources.

### 7.2. Network-Level Approaches

Advanced analytical approaches offer powerful tools for probing the network-level alterations that underlie CBS hallucinations. In particular, methods such as dynamic causal modeling and related techniques that infer directed interactions between brain regions may help clarify changes in effective connectivity within the visual system and beyond. By characterizing the directionality of information flow, these approaches could reveal whether hallucinations primarily reflect aberrant bottom-up signaling, disinhibited top-down influences, or a combination of both, thereby providing a direct empirical test of central predictions derived from predictive processing accounts of hallucination generation. Graph-theoretical analyses provide a complementary perspective by enabling quantitative characterization of large-scale network topology and information flow within visual processing hierarchies. Metrics capturing network segregation, integration, and small-world properties may reveal how alterations in connectivity patterns relate to specific hallucinatory features. For example, differences in network organization may distinguish patients who experience complex, semantically rich hallucinations from those whose experiences are limited to simpler visual phenomena, offering insights into how network topology constrains perceptual content.

Finally, multimodal integration approaches that combine structural, functional, and neurochemical measures hold particular promise for developing comprehensive models of CBS pathophysiology. By integrating indices of structural connectivity derived from diffusion imaging with functional dynamics measured through fMRI or EEG, alongside neurochemical profiles assessed using magnetic resonance spectroscopy, future studies could elucidate how multiple interacting factors converge to produce hallucinations in individual patients. Such integrative frameworks would move the field beyond single-modality explanations and toward a more complete understanding of the network-level mechanisms that give rise to CBS hallucinations.

### 7.3. Precision Medicine Approaches

The marked heterogeneity observed in CBS presentations highlights the potential value of subtype identification approaches. Determining whether distinct neurobiological mechanisms underlie different hallucinatory phenomenologies, such as simple versus complex experiences, colored versus achromatic imagery, or static versus dynamic percepts, could substantially refine both theoretical models and clinical management strategies. Addressing this question will require studies involving larger and well-characterized cohorts of CBS patients, combining standardized assessments of hallucination features with detailed neuroimaging and electrophysiological measures. Such efforts would allow researchers to move beyond descriptive classifications and toward biologically informed subtypes of CBS. Closely related to this goal is the development of reliable biomarkers; this represents a critical step toward more personalized treatment approaches. Identifying neurophysiological or neuroimaging markers that predict treatment response could guide clinical decision-making and reduce reliance on trial-and-error strategies. For instance, specific EEG signatures might differentiate patients more likely to benefit from acetylcholinesterase inhibitors as opposed to antipsychotic medications, while patterns of functional connectivity could help identify individuals who are most likely to respond to non-invasive brain stimulation techniques.

Ultimately, these precision-oriented approaches could enable genuinely targeted interventions tailored to individual patterns of neural dysregulation. Rather than applying uniform treatments across a heterogeneous patient population, clinicians could select pharmacological agents, stimulation parameters, or behavioral interventions based on objectively measured neural characteristics. Such personalized medicine strategies have the potential to improve therapeutic outcomes while simultaneously minimizing unnecessary medication exposure and associated side effects.

### 7.4. Translational Research

Computational modeling provides a powerful framework for integrating the diverse empirical findings reviewed here and for generating testable predictions about the mechanisms underlying CBS. Further development of biologically plausible models that simulate how alterations in neural circuits give rise to hallucinatory percepts could help bridge the gap between cellular-level changes and subjective perceptual experience. Importantly, such models could be fitted to individual patient data, potentially revealing mechanistic differences across CBS subtypes and offering principled guidance for the selection of targeted interventions.

Although animal models cannot capture the subjective phenomenology of hallucinations, ethologically relevant models of sensory deafferentation nonetheless offer valuable opportunities to investigate the cellular and molecular processes that accompany adaptation to visual loss. These approaches could clarify specific changes in synaptic function, neurotransmitter signaling, and large-scale network dynamics that follow deafferentation, thereby identifying novel biological targets for therapeutic intervention.

At the translational level, there is a growing need for mechanism-based clinical trials that focus on specific aspects of neural dysregulation rather than on symptom suppression alone. Designing interventions around hypothesized mechanisms, such as restoring excitation–inhibition balance, enhancing cholinergic modulation of sensory precision, or stabilizing network dynamics, would allow clinical trials to simultaneously test mechanistic predictions and evaluate therapeutic efficacy. Incorporating biomarkers linked to the targeted mechanism would further strengthen this approach, enabling researchers to assess target engagement directly and to determine whether modulation of the proposed neural process is associated with meaningful clinical improvement.

Despite significant progress in understanding CBS, several aspects of the integrated model presented here remain hypothetical or based on inference from indirect data. The application of predictive processing frameworks to CBS, while theoretically compelling, awaits more direct empirical validation. Similarly, the precise roles of specific neurotransmitter systems in modulating the balance between bottom-up and top-down processing in CBS require further investigation with techniques that can capture dynamic neurochemical changes during hallucinatory episodes. These limitations highlight the need for studies specifically designed to test key predictions of the integrated model, particularly those examining the relationship between neural desynchronization and hallucination phenomenology. Such work would strengthen the empirical foundation of the theoretical framework advanced in this review.

## 8. Conclusions

Charles Bonnet syndrome offers a uniquely informative window into the neural mechanisms underlying visual perception and conscious experience. The evidence reviewed here indicates that CBS hallucinations do not arise from simple hyperexcitability of the deafferented visual cortex, but rather from complex alterations in the dynamics of distributed neural networks supporting visual processing. By situating CBS within an integrated framework that spans sensory constraints, neural implementation, and computational inference, this review highlights how hallucinations emerge from dynamic interactions across multiple levels of explanation, underscoring the necessity of multiscale approaches for both understanding and treating this condition. Central to this framework is the desynchronization between bottom-up sensory signals and top-down predictive processes, further modulated by neurotransmitter systems and contextual factors. Visual deafferentation establishes the initial conditions by reducing the precision of sensory input, release mechanisms shape the emergence of structured internal representations, and failures of predictive inference explain how these internally generated representations are misattributed to external reality rather than recognized as endogenous perceptual activity. This integrated perspective reconciles several apparently contradictory findings in the literature and provides a coherent account of key clinical features of CBS. It helps explain why only a subset of visually impaired individuals develop hallucinations, why hallucinatory content is often semantically rich and structured, and why hallucinations tend to occur episodically rather than as a continuous perceptual state. Moreover, it accounts for the marked heterogeneity in treatment response, suggesting that different therapeutic approaches may engage distinct components of a multilevel pathophysiology rather than a single underlying mechanism. It should be emphasized that the therapeutic implications discussed above are largely mechanistically inspired and not yet supported by robust large-scale clinical trials. Current treatment approaches remain empirical and exploratory, underscoring the need for adequately powered randomized studies. Beyond its specific relevance to CBS, the neural desynchronization framework advanced here has broader implications for understanding hallucinatory phenomena across clinical and non-clinical contexts. More generally, it offers insights into the neural basis of perception and consciousness, illustrating how perceptual experience emerges from the ongoing negotiation between sensory evidence and prior expectations and how disruptions to this balance can profoundly alter the experience of reality. As experimental tools and analytical methodologies continue to advance, our understanding of CBS is likely to evolve further, opening the door to more refined diagnostic strategies and mechanism-targeted interventions. Progress in this field will depend on research that fully embraces the complexity of neural systems across multiple levels of explanation, from molecular and cellular mechanisms to network dynamics and computational principles. Ultimately, continued investigation of CBS promises not only to improve clinical care for affected individuals but also to deepen our fundamental understanding of how the brain constructs visual reality and what happens when these constructive processes go awry.

## Figures and Tables

**Table 1:** The table summarizes major explanatory frameworks for CBS across levels of analysis, highlighting how the proposed multilevel model integrates reduced sensory precision, homeostatic plasticity, selective disinhibition, and neural desynchronization to extend prior accounts.

Model	Level of Explanation	Core Mechanism	Explanatory Scope
Deafferentation Hypothesis	Implementation	Loss of visual input reduces the precision and reliability of bottom-up signals	Explains localization of hallucinations to areas of visual loss, but not semantic richness or episodic nature
Release Phenomenon Theory	Implementation	Homeostatic plasticity, gain changes, and selective disinhibition amplify internally generated representations after deprivation	Explains latency after vision loss and the emergence of structured hallucination content
Perception and Attention Deficit (PAD) Model	Algorithmic	Impaired sensory processing combined with dysfunctional attentional binding	Explains complex hallucinations via attentional-perceptual mismatch, but does not specify the underlying neural implementation
Predictive Processing Accounts	Computational	Reduced precision-weighting of sensory prediction errors shifts inferential balance toward top-down priors	Explains semantic organization and episodic fluctuations through inferential imbalance
Proposed Integrated Model	Multilevel (Computational, Algorithmic, Implementation)	Reduced sensory precision + homeostatic plasticity + selective disinhibition + desynchronization between bottom-up and top-down processing disrupts hierarchical coordination	Accounts for semantic richness, episodic occurrence, preserved insight, weak correlation between severity of visual impairment and hallucination complexity, and heterogeneous treatment responses across individuals.

**Table 2:** The table outlines how different pathophysiological accounts could guide distinct management strategies, emphasizing the integrative scope of the proposed multilevel model.

Model	Clinical Implications
Deafferentation Hypothesis	Emphasizes optimization of residual vision through visual rehabilitation, correction of ocular pathology, and enhancement of bottom-up sensory input
Release Phenomenon Theory	Suggests potential benefit of interventions targeting cortical plasticity and excitability regulation, including neuromodulatory approaches
Perception and Attention Deficit (PAD) Model	Supports psychological and attentional interventions aimed at improving perceptual-attentional integration and reducing distress
Predictive Processing Accounts	Encourages strategies restoring balance between bottom-up evidence and top-down expectations, including cholinergic modulation and psychological approaches
Proposed Integrated Model	Supports multimodal, mechanism-based management combining visual rehabilitation, patient education, targeted neuromodulation, and individualized pharmacotherapy

**Table 3:** Evidence levels are based on the Grading of Recommendations Assessment, Development and Evaluation (GRADE) criteria: Moderate = single small RCT (N = 12) or observational study with consistent, clinically meaningful findings; Low = observational studies with limited replication; Very low = case reports or small case series without controls.

Intervention	Study Design	Level of Evidence	Main Limitations	Reference(s)
Non-Pharmacological interventions				
Patient education and reassurance	Observational study	Moderate	Subjective outcomes; no RCTs;	Cox and Ffytche, 2014 [47]
Visual rehabilitation (corrective lenses, low-vision aids, treatment of ocular pathology)	Observational studies	Low	No controlled trials; heterogeneous interventions; modest effects on hallucination frequency	Schadlu et al., 2009; Menon et al., 2003 [6,14]
Environmental modifications (improved lighting, reduced visual clutter, multisensory stimulation)	Anecdotal reports	Low	No systematic evaluation or controlled studies	Menon et al., 2003 [14]
Behavioral coping strategies (eye movements, blinking, attention redirection, verbal addressing)	Patient self-reports	Low	No controlled studies; subjective effectiveness only	Jones et al., 2021 [7]
Neuromodulation				
Transcranial direct current stimulation (tDCS)—anodal occipital	Randomized placebo-controlled crossover trial	Moderate	Small sample size (N = 12); single RCT; needs replication in larger cohorts	daSilva Morgan et al., 2022 [49]
Pharmacological interventions				
Atypical antipsychotics (olanzapine, quetiapine)	Case series (N = 3)	Very low	Open-label; no controls; significant side effect concerns in the elderly population	Coletti Moja et al., 2005 [50]
Acetylcholinesterase inhibitors (donepezil, rivastigmine)	Single case reports	Very low	No controlled trials; unclear generalizability	Ukai et al., 2004 [35]
Selective serotonin reuptake inhibitors (SSRIs) and serotonin-norepinephrine reuptake inhibitors (SNRIs)	Case series (N = 21, N = 4)	Very low	Open-label; no placebo control; retrospective data; mechanism unclear	Lang et al., 2007; Bergman and Barak, 2013 [40,48]
Anticonvulsants (gabapentin, carbamazepine, valproate)	Single case report (N = 1)	Very low	No replication studies; no controlled trials	Paulig and Mentrup, 2001 [51]
Benzodiazepines	Observational reports	Very low	No controlled trials; no reduction in hallucination frequency; sedating properties and cognitive side effects limit utility in elderly patients	Teunisse et al., 1996; Menon et al., 2003 [13,14]

## Data Availability

No new data were created or analyzed in this study. Data sharing is not applicable to this article.

## Abbreviations

The following abbreviations are used in this manuscript:

AMD	Age-related macular degeneration
CBS	Charles Bonnet syndrome
DBM	Deep Boltzmann machine
DMN	Default mode network
EEG	Electroencephalography
fMRI	Functional magnetic resonance imaging
GRADE	Grading of Recommendations Assessment, Development and Evaluation
ICD-11	International Classification of Diseases
MEG	Magnetoencephalography
TMS	Transcranial Magnetic Stimulation
tDCS	Transcranial direct current stimulation
SNRIs	Serotonin-norepinephrine reuptake inhibitors
SSRIs	Selective serotonin reuptake inhibitors
